# The OpenWorm Project: currently available resources and future plans

**DOI:** 10.1186/1471-2202-16-S1-P141

**Published:** 2015-12-18

**Authors:** Padraig Gleeson, Matteo Cantarelli, Michael Currie, Jim Hokanson, Giovanni Idili, Sergey Khayrulin, Andrey Palyanov, Balazs Szigeti, Stephen Larson

**Affiliations:** 1 Department of Neuroscience, Physiology and Pharmacology, University College London, London, UK; 2 OpenWorm Foundation, 3899 Nobel Drive, Unit 1226, San Diego, CA 92122, USA; 3 Department of Biomedical Engineering, Duke University, Durham, NC, USA; 4 Lab. of Complex Systems Simulations, A.P. Ershov Institute of Informatics Systems, Siberian Branch of the Russian Academy of Sciences, Novosibirsk, 630090, Russian Federation; 5 Neuroinformatics Doctoral Training Centre, University of Edinburgh, Edinburgh, UK

## 

The nematode C. elegans is historically one of the most studied model organism in biology and the only one to have its full connectome mapped. OpenWorm [1] is a project dedicated to recreating the C. elegans nematode as a virtual organism in a computer. The project takes an Open Science approach to development, relying on volunteer contributions and making all code, data and documentation publicly available at the time of production. The OpenWorm project has two long term goals. The first is to functionally reproduce the behaviour of the wild-type C. elegans in a variety of environmental contexts, to the extent that the simulated behaviour is statistically indistinguishable from recordings of real worms under analogous environmental conditions. OpenWorm's second goal is for the simulation to be a faithful biological model for C. elegans. The achievement of these ambitious goals will hopefully make OpenWorm a valuable software tool in C. elegans labs worldwide. Scientists could make perturbations *in-silico *before beginning the expensive and time-consuming work of conducting *in-vivo *experiments. Conversely, having many scientist users will engender a feedback process that will make development more data-driven, helping to improve the biological realism of the OpenWorm model in the first place.

The medium term aim of the project is to create infrastructure to enable these longer term goals and tackle smaller but significant challenges in whole organism modelling along the way. The first concrete milestone the project has set for itself is an accurate simulation of the mechanical activity of the worm including the motor system, with accurate electrophysiology of the C. elegans muscle cells [2] to reproduce the crawling gait. This model will be validated by comparing crawling behaviour from experimental data. We aim to make this model accessible through a web browser so that scientists and even interested members of the public can explore and experiment with the model in 3D.

A number of activities are ongoing towards achieving this milestone: **Sibernetic: **a Smoothed Particle Hydrodynamics based simulation engine to simulate the environment and the body of the worm, with the ability to simulate fluids of variable viscosities and contractible elastic matter with impermeable membranes; **Geppetto: **a web based visualization and simulation engine being developed to bring the output of the OpenWorm project to the web for maximum accessibility, without the need for local installation of the software required to run simulations of the model; **c302: **a framework based on NeuroML for generating network models of C. elegans incorporating known anatomical data which can models for the neurons of varying levels of detail (point neurons to multicompartmental); **PyOpenWorm**, a Python based API for accessing the latest anatomical and physiological data on C. elegans, facilitating its use in computational models, with data provenance built in from the start; **Movement Validation Framework: **a suite of tools for analyzing worm behaviour allowing extraction from videos (or from simulated activity) of known motifs of worm movement.

All of the above applications are open source and can be reused for modelling other species/brain regions. More details can be found on http://www.openworm.org and https://github.com/openworm.
